# The impact of genomic selection on genetic diversity and genetic gain in three French dairy cattle breeds

**DOI:** 10.1186/s12711-019-0495-1

**Published:** 2019-09-23

**Authors:** Anna-Charlotte Doublet, Pascal Croiseau, Sébastien Fritz, Alexis Michenet, Chris Hozé, Coralie Danchin-Burge, Denis Laloë, Gwendal Restoux

**Affiliations:** 1 GABI, INRA, AgroParisTech, Université Paris-Saclay, 78350 Jouy-en-Josas, France; 2 ALLICE, Paris, France; 3 Institut de l’Elevage, Paris, France

## Abstract

**Background:**

In France, implementation of genomic evaluations in dairy cattle breeds started in 2009 and this has modified the breeding schemes drastically. In this context, the goal of our study was to understand the impact of genomic selection on the genetic diversity of bulls from three French dairy cattle breeds born between 2005 and 2015 (Montbéliarde, Normande and Holstein) and the factors that are involved.

**Methods:**

We compared annual genetic gains, inbreeding rates based on runs of homozygosity (ROH) and pedigree data, and mean ROH length within breeds, before and after the implementation of genomic selection.

**Results:**

Genomic selection induced an increase in mean annual genetic gains of 50, 71 and 33% for Montbéliarde, Normande and Holstein bulls, respectively, and in parallel, the generation intervals were reduced by a factor of 1.7, 1.9 and 2, respectively. We found no significant change in inbreeding rate for the two national breeds, Montbéliarde and Normande, and a significant increase in inbreeding rate for the Holstein international breed, which is now as high as 0.55% per year based on ROH and 0.49% per year based on pedigree data (equivalent to a rate of 1.36 and 1.39% per generation, respectively). The mean ROH length was longer for bulls from the Holstein breed than for those from the other two breeds.

**Conclusions:**

With the implementation of genomic selection, the annual genetic gain increased for bulls from the three major French dairy cattle breeds. At the same time, the annual loss of genetic diversity increased for Holstein bulls, possibly because of the massive use of a few elite bulls in this breed, but not for Montbéliarde and Normande bulls. The increase in mean ROH length in Holstein may reflect the occurrence of recent inbreeding. New strategies in breeding schemes, such as female donor stations and embryo transfer, and recent implementation of genomic evaluations in small regional breeds should be studied carefully in order to ensure the sustainability of breeding schemes in the future.

## Background

In France, genomic evaluations started to be implemented in dairy cattle breeds in 2009. After a brief transition period for the breeding companies to adopt this technology, these new evaluation methods have drastically changed dairy cattle breeding schemes since 2011. Genomic evaluations have allowed breeding companies to increase the number of male candidates for selection and the use of new bulls [1, 2]. At the early selection stage, genomic selection (GS) allows exploiting a major part of the genetic variance compared to the previously used models based on parents’ average values [1, 3]. In fact, Mendelian sampling variation, which is defined as the difference between the genetic value of an individual and the average of the parental genetic values, can be captured from molecular markers without any information on the parents’ phenotype or the offspring’s phenotype. This makes it possible to obtain breeding values with higher reliability sooner in the life of an individual compared with those based on pedigree data [4]. Indeed, the estimation of pedigree-based breeding values at birth depends mostly on information about related individuals, resulting in a low reliability and a high correlation between estimated breeding values of relatives. These high correlations decrease only later in the life of an animal when information based on its own performance or performances of the progeny becomes available. Consequently, GS reduces the number of related individuals chosen as candidates for selection [5]. Moreover, collecting genotype data is less expensive than collecting phenotype data, which means that more male candidates can be evaluated with high reliability. Furthermore, methods to balance inbreeding and genetic gain in breeding schemes, such as optimal contribution selection [6], allow for better conservation of genetic diversity when based on genomic information rather than on pedigree data [7, 8]. Thus, GS was expected to reduce the rate of inbreeding and to increase genetic gain per generation at the same time [2].

However, since animals with high genomic estimated breeding values (GEBV) can be selected as parents at a very young age, generation intervals are significantly shortened [9]. As a result, the annual inbreeding rate could then increase and this has already been observed in Dutch-Flemish Holstein–Friesian cattle [10] and in North American Holstein cattle [11].

Such an increase in inbreeding rate results in a loss of genetic diversity, with lower additive genetic variance leading to a lower response to selection [12]. Moreover, a lower overall genetic variability leads also to a loss of adaptive potential for selecting new breeding goals in the context of climate change [13], and more severe inbreeding depression [14–16].

Measuring genetic diversity is necessary in order to manage it. Typically, genetic diversity is estimated and managed with the help of inbreeding and kinship coefficients based on pedigree data (e.g. [17, 18]). These coefficients reflect the proportion of genome expected to be identical-by-descent (IBD) in an individual and between two individuals, respectively. The reliability of these estimates depends on the quality of the pedigree records and their depth [7, 19, 20]. In particular, pedigree-based inbreeding and kinship may be underestimated due to incomplete pedigree data and the assumption of arbitrary non-inbred and unrelated founder individuals, thus leading to an overestimation of genetic diversity.

Falling costs in the field of high-throughput genotyping and sequencing make it possible to obtain more and more genotyping data, and thus to estimate genetic diversity more precisely. These data allow the computation of inbreeding coefficients and genomic relationship matrices (GRM) [21, 22]. The calculation of inbreeding coefficients from GRM computed using all the allele frequencies set to 0.5 globally consists in computing observed and expected proportions of homozygous single nucleotide polymorphisms (SNPs) considered as identical-by-state (IBS).

Another method to estimate genetic diversity consists in detecting IBD segments, called runs of homozygosity (ROH), by considering that segments of consecutive homozygous SNPs originate from common ancestors [23, 24]. Thus, it is possible to compute ROH-based inbreeding coefficients defined as the proportion of the autosomal genome that is actually included in ROH above a given length threshold [24], corresponding to the realized proportion of genome that is IBD. This approach to estimate inbreeding coefficients seems to reflect IBD better than genomic inbreeding coefficients calculated from molecular data based on the GRM [11, 25–27]. The length of such ROH segments follows an inverse exponential distribution with expectation 1/2G Morgan, with G being the number of generations to the common ancestor from which the segment arises (e.g. [28]). Due to recombination, ROH length tends to decrease over generations [29, 30], which allows to estimate the age of inbreeding events along the genome and to trace back population history [29, 31, 32]. Shorter ROH tend to result from older inbreeding (older common ancestors) whereas longer ROH tend to result from more recent inbreeding (more recent common ancestors) [29, 31, 32]. However, different criteria have been proposed to define ROH-based inbreeding estimates and there is no consensus about which one should be preferred (e.g. the number of heterozygous loci allowed in one ROH, the minimal length, etc.). Consequently, ROH-based inbreeding estimates might differ, depending on the criteria used [28].

Pedigree data are useful to evaluate genetic diversity for individuals that are not genotyped, whereas measures based on molecular data (such as ROH) allow the study of the realized proportion of genome that is autozygous and the pattern of inbreeding along the genome. In spite of their advantage over pedigree-based inbreeding estimates, genomic-based estimates began to be used to manage genetic diversity in selection programs only recently [33–35].

In this context, the goal of our study was to understand the impact of GS on genetic diversity in three French dairy cattle breeds: Montbéliarde and Normande, two national breeds, and Holstein, an international breed. We measured the relative changes in rates of genetic merit and in three different estimates of genetic diversity: (i) pedigree-based inbreeding and kinship, (ii) ROH-based inbreeding coefficients, and (iii) mean length of ROH; and these estimates were taken before and after the implementation of GS in these three breeds in France. Based on our findings, we compare pedigree and ROH-based inbreeding rates within breeds and explore the changes in breeding schemes that could explain the differences observed between national and international breeds.

## Methods

### Animals, pedigree and genotyping data

**Fig. 1:**
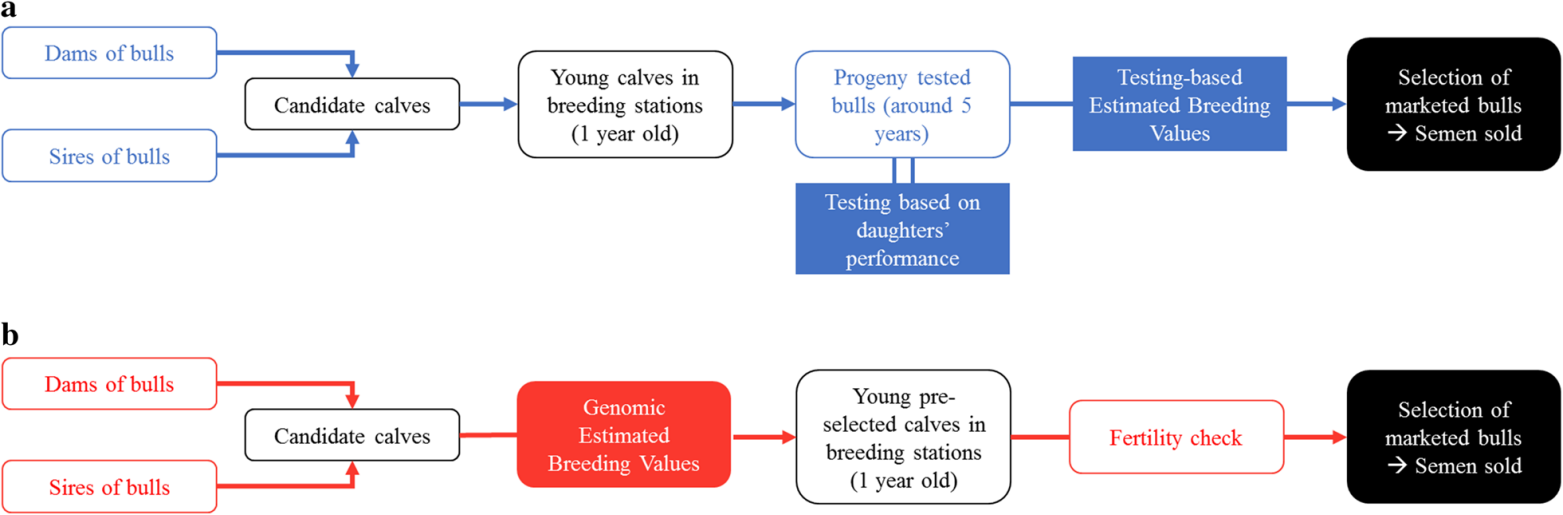
Breeding schemes of bulls under progeny testing (**a**) and genomic (**b**) selection

We used three datasets that consisted of all progeny-tested bulls born between 2005 and 2010 and all marketed bulls born between 2012 and 2015 from three French dairy cattle breeds: 1307 Montbéliarde bulls and 1016 Normande bulls (national breeds), and 4694 Holstein bulls (international breed) (see Table 1 and Fig. 1). We chose these categories of bulls in order to obtain cohorts of comparable sizes and to compare bulls that are selected for artificial insemination by breeding companies (either to be marketed or to be tested). Key data about these breeds in France are available in Additional file 1: Table S1.

**Table 1:** Number of animals in each dataset

Breed	Birth period	Number of individuals born each year				Total number of individuals
		Min	Mean (± sd)	Max	Median	
Montbéliarde	2005–2010 Progeny-tested bulls	82	138 (± 34)	176	141	826
	2012–2015 Marketed bulls	81	120 (± 29)	149	125	480
Normande	2005–2010 Progeny-tested bulls	82	111 (± 21)	135	112	666
	2012–2015 Marketed bulls	73	87 (± 11)	100	87	347
Holstein	2005–2010 Progeny-tested bulls	368	573 (± 157)	697	606	3440
	2012–2015 Marketed bulls	294	312 (± 12)	322	316	1248

Genealogies of all bulls in the three datasets were traced back as far as possible. The mean numbers of generations traced back for bulls in the datasets were 15.4 ± 1.7, 15.8 ± 1.6 and 15.4 ± 1.9 for Montbéliarde, Normande and Holstein, respectively (longest paths computed with R [36] package pedigree [37]).

All the bulls in the datasets were genotyped for 50 K SNPs (Illumina Infinium^®^ BovineSNP50 BeadChip). Quality control filtering was performed by removing SNPs that were genotyped in less than 95% individuals and SNPs with a minor allele frequency (MAF) lower than 1%. No individual had less than 95% genotyped SNPs. After quality control filtering, 39,992, 40,135 and 41,377 SNPs remained in the datasets for Montbéliarde, Normande and Holstein, with on average one SNP every 62.5 ± 66.5 kb, 62.2 ± 66.4 kb and 60.4 ± 63.8 kb, respectively.

The total genetic merit index ISU (standing for *Index Synthèse Unique*) is an individual index that combines production, functional and type traits, which are weighted depending on the breed and subsequent breeding goals. The formula to obtain total genetic merit index ISU is built in order to obtain a standard error of the index of 20 points within each breed [38]. Within each breed, all genetic merit estimates were calculated relative to the same arbitrary base.

### Measures of genetic diversity

#### Pedigree-based measures of genetic diversity

Pedigree-based inbreeding coefficients $F_{\text{ped}}$ and kinship coefficients $f_{\text{ped}}$ were computed with the Pedig software [39] for all the animals in the three datasets. The mean of all kinship coefficients $f_{\text{ped}}$ for bulls born between 2005 and 2010 and the mean of all kinship coefficients $f_{\text{ped}}$ for bulls born between 2012 and 2015 were compared with a two-sample *t* test. We also computed pedigree-based coefficients by taking only the last five generations for each bull into account, with the Pedig software [39] for all animals in the three datasets.

#### Measures of genetic diversity based on runs of homozygosity

ROH reflect autozygous segments of the genome. A ROH was defined as a homozygous segment of at least 15 SNPs or 1000 kb long, with at least one SNP per 75 kb. Two consecutive SNPs could not be included in the same ROH if they were more than 150 kb apart. ROH were detected using the PLINK 1.9 “homozyg” function [40, 41] (command line: plink --cow --bfile genotyping_data_filename --homozyg --homozyg-kb 1000 --homozyg-snp 15 --homozyg-window-snp 15 --homozyg-density 75 --homozyg-gap 150 --out output_filename).

ROH-based inbreeding estimates, $F_{{{\text{ROH,}}i}}$, were computed as the proportion of genome included in ROH [10, 24] as follows:

$$F_{{{\text{ROH,}}i}} = \frac{{\varSigma L_{{{\text{ROH}},i}} }}{{L_{\text{auto}} }},$$

where $\varSigma L_{{{\text{ROH}},i}}$ is the total length of ROH for individual $i$, and $L_{\text{auto}}$ the length of the autosomal genome covered by SNPs after withholding gaps longer than 150 kb between two SNPs, corresponding to the length of the autosomal genome on which ROH can be detected. These parameters allowed the detection of ROH on 77.0, 77.0 and 78.6% of the autosomal genome of Montbéliarde, Normande and Holstein bulls, respectively (see Additional file 2: Table S2).

For each individual, the mean ROH length, i.e. $L_{{{\text{ROH}},{\text{mean}},i}}$, was computed as:

$$L_{{{\text{ROH}},{\text{mean}},i}} = \frac{{\varSigma L_{{{\text{ROH}},i}} }}{{N_{{{\text{ROH}},i}} }},$$

where $\varSigma L_{{{\text{ROH}},i}}$ is the total length of ROH for individual $i$ in kb, and $N_{{{\text{ROH}},i}}$ the total number of ROH for individual $i$.

Pearson’s product-moment correlations between pedigree- and ROH-based inbreeding coefficients were computed for each breed in order to check if it was relevant to compare both inbreeding estimates.

To compare the variability of pedigree and ROH-based inbreeding rates, we calculated the coefficient of variation of these two parameters within each breed as the ratio between the overall standard deviation of inbreeding and its overall mean for each dataset.

We obtained approximate inbreeding rates per generation for a selection type by multiplying the mean inbreeding rate by the mean parental generation interval for this period (in years) (see Additional file 3: Table S3).

### Demographic parameters

Generation interval $I_{1}$ was computed with an adapted version of Pedig software [39]. $I_{1}$ was defined as the mean difference between the birthdate of an individual and its parents, in months.

For each breed, the number of bulls born per year was divided by the number of bulls born in 2005 in the three datasets, giving $S_{i}$:

$$S_{i} = \frac{{N_{i} }}{{N_{2005} }},$$

where $N_{i}$ is the number of bulls born in year $i$.

The effective number of bulls, $Ne_{\text{breed, selection}}$, for a given breed and a given type of selection was computed as:

$$Ne_{\text{breed, selection}} = \frac{1}{{\sum\nolimits_{i = 1}^{{n\,_{{{\text{breed,}}\,{\text{selection}}}} }} {\left( {\frac{{o_{i} }}{{\sum\nolimits_{j = 1}^{{n\,_{{{\text{breed,}}\,{\text{selection}}}} }} {o_{j} } }}} \right)^{2} } }},$$

where $n_{\text{breed, selection}}$ is the number of bulls for a given breed and a given type of selection and $o_{i}$ the number of offspring of bull $i$ [42, 43]. We also computed the effective/census number of bulls ratio, ${{Ne_{\text{breed, selection}} } \mathord{\left/ {\vphantom {{Ne_{\text{breed, selection}} } {n_{\text{breed, selection}} }}} \right. \kern-0pt} {n_{\text{breed, selection}} }}$. The two types of selection were progeny testing selection (PTS) (individuals born between 2005 and 2010), and genomic selection (GS) (individuals born between 2012 and 2014).

95% confidence intervals for $Ne_{\text{breed, selection}}$ and ${{Ne_{\text{breed, selection}} } \mathord{\left/ {\vphantom {{Ne_{\text{breed, selection}} } {n_{\text{breed, selection}} }}} \right. \kern-0pt} {n_{\text{breed, selection}} }}$ were generated by random resampling with replacement of the datasets (bootstrap with 1000 iterations). For a given breed, $Ne_{\text{breed, PTS}}$ and $Ne_{\text{breed, GS}}$, or ${{Ne_{\text{breed, PTS}} } \mathord{\left/ {\vphantom {{Ne_{\text{breed, PTS}} } {n_{\text{breed, PTS}} }}} \right. \kern-0pt} {n_{\text{breed, PTS}} }}$ and ${{Ne_{\text{breed, GS}} } \mathord{\left/ {\vphantom {{Ne_{\text{breed, GS}} } {n_{\text{breed, GS}} }}} \right. \kern-0pt} {n_{\text{breed, GS}} }}$ were statistically different if confidence intervals did not overlap.

### Impact of genomic selection on genetic diversity and genetic gain

In order to assess the impact of GS on genetic diversity and genetic gain, we used the following linear model, using the R function lm [36]:

$$Y_{i} = \left\{ {\begin{array}{*{20}c} {a_{1} + b_{1} .x_{i} + \varepsilon_{i} , 2005 \le x_{i} \le 2010} \\ {a_{2} + \left( {b_{1} + \delta } \right).x_{i} + \varepsilon_{i} , 2012 \le x_{i} \le 2015} \\ \end{array} } \right.$$

where $Y_{i}$ is the variable of interest for bull $i$ ($F_{\text{ped}}$, $f_{\text{ped}}$, $F_{{{\text{ROH,}}i}}$, $L_{{{\text{ROH}},{\text{mean}},i}}$ for genetic diversity and ISU for genetic gain), $x_{i}$ is the birth year of bull $i$, and $b_{1}$ the associated coefficient of regression if bull $i$ was born between 2005 and 2010 (PTS) or $\left( {b_{1} + \delta } \right)$ if born between 2012 and 2015 (GS). The impact of GS was measured with the $\delta$ coefficient. Its significance was tested with an analysis of variance.

The quality of linear regressions was checked by looking at the square root of the standardized residuals of each linear regression (see Additional file 4: Figures S1–S7).

The relative change, $RC$, of the slopes of regression before and after GS was computed as:

$$RC = \frac{\delta }{{\left| {b_{1} } \right|}}.$$

For a better interpretation of relative change, please note that the sign of *RC* quantifies the direction of the change of the slopes (if *RC* > 0, the new slope will be higher than the previous slope and if *RC* < 0, it will be lower) and its absolute value quantifies the magnitude of the change of the slopes.

## Results

### Genetic merit

**Fig. 2:**
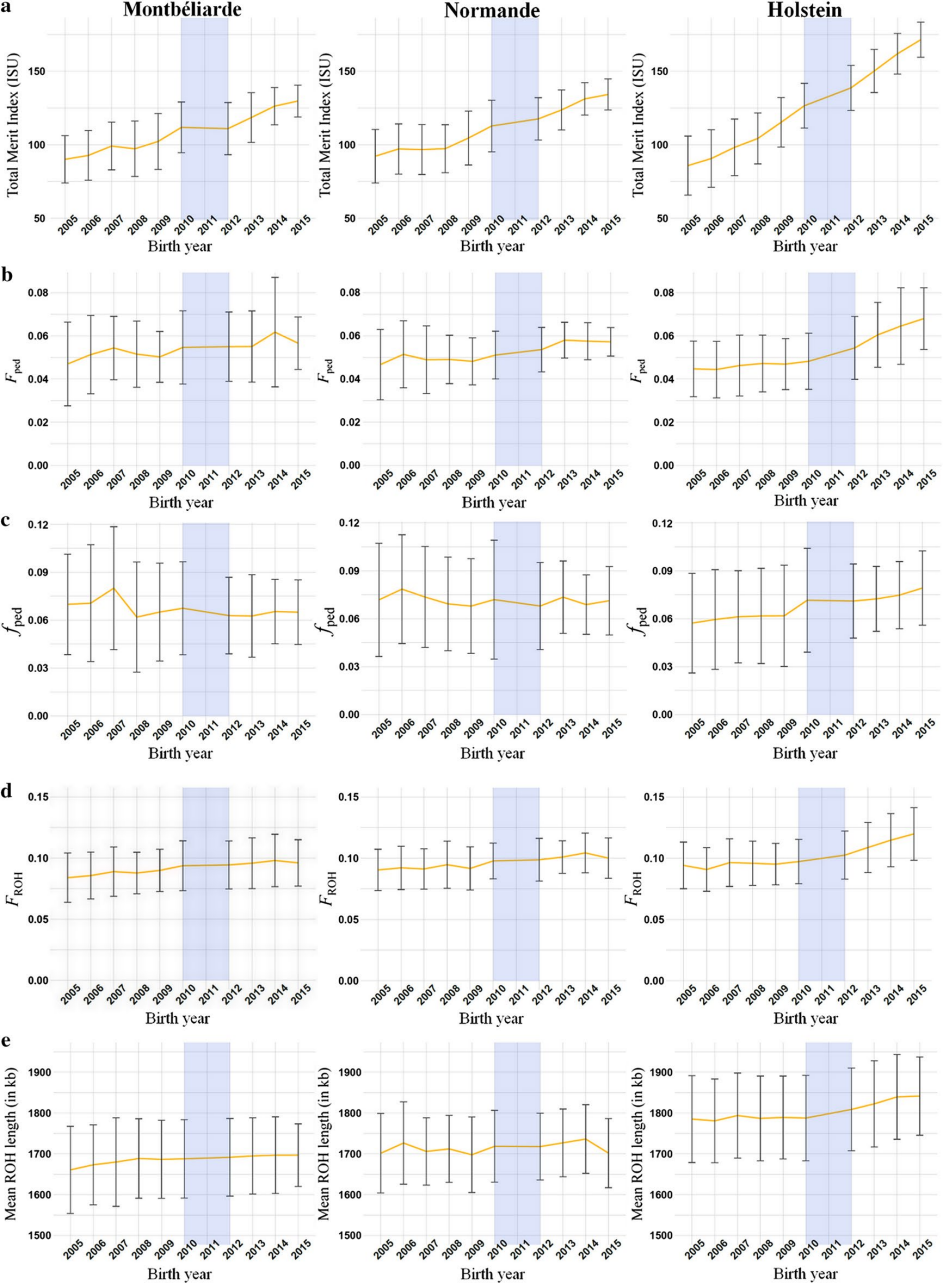
Trends of the different parameters estimating genetic gain and genetic diversity. The yellow line represents the mean for each birth year and the error bars the standard deviation. Total merit indices (ISU) (**a**), inbreeding (**b**) and kinship (**c**) based on pedigree data, inbreeding based on runs of homozygosity (ROH) (**d**) and mean ROH length (in kb) (**e**) were retrieved or computed for progeny-tested bulls born between 2005 and 2010 and for marketed bulls born between 2012 and 2015, for Montbéliarde, Normande and Holstein

The total genetic merit index ISU increased at a significantly higher rate in Montbéliarde, Normande and Holstein under GS (between 2012 and 2015) than under PTS (between 2005 and 2010), with respective relative changes of 0.50, 0.71 and 0.33 (see Table 2 and Fig. 2a). The slopes of ISU during GS are equivalent to increases of ~ 4.4, 5.0 and 7.4% per year relative to the mean genetic merit ISU in 2012 of 111, 118 and 139 points for Montbéliarde, Normande and Holstein, respectively.

**Table 2:** Trends of the different inbreeding criteria estimating genetic gain and genetic diversity per year

Parameter	Breed	*b*_1_ (± SE)	*b*_*2*_	$\varvec{\delta}$ (± SE)	*p*-value of $\varvec{\delta}$	RC
ISU	MON	3.25 (± 0.28)	4.89	1.64 (± 0.72)	0.023	0.50
	NOR	3.42 (± 0.31)	5.85	2.43 (± 0.79)	2.1e−03	0.71
	HOL	7.77 (± 0.16)	10.31	2.54 (± 0.41)	6.9e−10	0.33
*F*_ped_ (in %)^a^	MON	0.090 (± 0.029)	0.11	0.019 (± 0.076)	0.81	0.21
	NOR	0.059 (± 0.024)	0.19	0.13 (± 0.061)	0.035	2.20
	HOL	0.088 (± 0.013)	0.49	0.40 (± 0.033)	< 1e−10	4.50
*f*_ped_ (in %)^a^	MON	− 0.14 (± 0.006)	− 0.013	0.13 (± 0.018)	< 1e−10	0.91
	NOR	− 0.11 (± 0.008)	0.072	0.19 (± 0.022)	< 1e−10	1.64
	HOL	0.20 (± 0.002)	0.22	0.019 (± 0.005)	1.5e−04	0.092
*F*_ROH_ (in %)^a^	MON	0.17 (± 0.033)	0.076	− 0.095 (± 0.085)	0.26	− 0.56
	NOR	0.12 (± 0.033)	0.14	0.019 (± 0.082)	0.82	0.16
	HOL	0.080 (± 0.018)	0.55	0.47 (± 0.045)	< 1e−10	5.93
Mean ROH^b^ length	MON	4.75 (± 1.64)	0.09	− 4.66 (± 4.22)	0.27	− 0.98
	NOR	1.32 (± 1.74)	1.2	− 0.149 (± 4.36)	0.97	− 0.11
	HOL	1.53 (± 0.965)	13	11.9 (± 2.43)	1.1e−06	7.77

*b*_*1*_ is the slope of regression of each parameter depending on birth year for progeny-tested bulls born between 2005 and 2010 (progeny testing selection), and *δ* the difference between the slopes of regression of each parameter depending on birth year for progeny testing selection and for marketed bulls born between 2012 and 2015 (genomic selection), for Montbéliarde (MON), Normande (NOR) and Holstein (HOL). The relative change (RC) is equal to $\frac{\delta }{{\left| {b_{1} } \right|}}$. *b*_*2*_ is the slope of the parameter for marketed bulls born between 2012 and 2015 (genomic selection), equal to *b*_*1*_ + $\delta$. The p-value of $\delta$ corresponds to the significance of the non-nullity of *δ*

ISU, total merit index; *F*_ped_, pedigree-based inbreeding; *f*_ped_, pedigree-based kinship; *F*_ROH_, inbreeding based on Runs of Homozygosity (ROH)

^a^Slopes and standard errors are displayed in %. *p*-values and relative changes are not

^b^Runs of homozygosity

### Genetic diversity

#### Pedigree-based measures of genetic diversity

Pedigree-based inbreeding rates increased significantly in Normande and Holstein under GS schemes (between 2012 and 2015) compared to PTS (between 2005 and 2010), i.e. from 0.059 to 0.088% per year to 0.19 and 0.49% per year for Normande and Holstein, respectively (from 0.34 to 0.46% per generation to 0.70 and 1.39% per generation, respectively) with respective relative changes of 2.20 and 4.50 (see Table 2). This was not the case in Montbéliarde, for which the inbreeding rate per year between 2005 and 2010 was 0.090% (0.52% per generation) (see Table 2). In 2005, the overall pedigree-based inbreeding level in Holstein did not differ significantly from that in Montbéliarde and Normande (see Fig. 2b). However, in 2015, the pedigree-based inbreeding level was significantly higher in Holstein than in Montbéliarde (*p*-value < 1e−10) and Normande (*p*-value < 1e−10) (see Fig. 2b). The slopes of pedigree-based inbreeding coefficients during GS are equivalent to increases of ~ 2.0, 3.5 and 8.9% per year of the mean pedigree-based inbreeding coefficients in 2012 for Montbéliarde, Normande and Holstein, respectively.

**Fig. 3:**
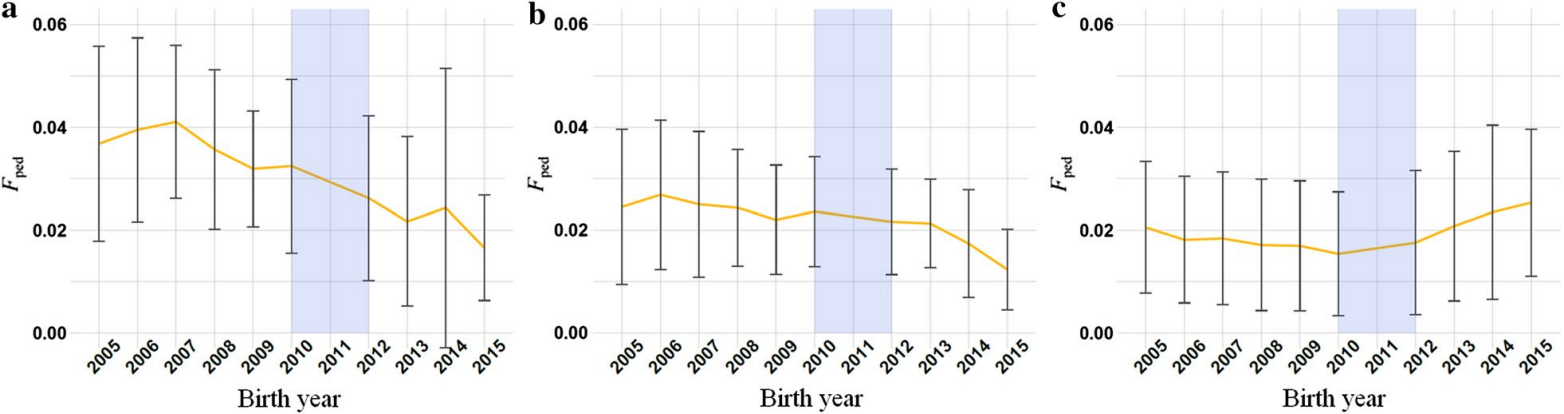
Pedigree-based inbreeding calculated from the last five generations. The yellow line represents the mean pedigree-based inbreeding calculated from the last five generations for each birth year and the error bars the standard deviation. Pedigree-based inbreeding calculated from the last five generations was computed for progeny-tested bulls born between 2005 and 2010 and for marketed bulls born between 2012 and 2015, for Montbéliarde (**a**), Normande (**b**) and Holstein (**c**)

For inbreeding coefficients based on pedigree and calculated over the last five generations only, the slope of regression was negative during PTS for the three breeds. Under GS, it decreased significantly for Montbéliarde and Normande, reaching − 0.32% and − 0.23% per year, respectively (− 1.22% and − 0.86% per generation, respectively), whereas it increased significantly for Holstein, reaching 0.27% per year (0.78% per generation) (Fig. 3) (see Additional file 5: Tables S4, S5).

Pedigree-based kinship increased at a significantly higher rate in Montbéliarde, Normande and Holstein under GS (between 2012 and 2015) than under PTS (between 2005 and 2010), with respective relative changes of 0.91, 1.64 and 0.092 (see Table 2). The slopes of pedigree-based kinship coefficients during GS are equivalent to increases of about − 0.20, 1.1 and 3.1% per year of the mean pedigree-based kinship coefficients in 2012 for Montbéliarde, Normande and Holstein, respectively (see Fig. 2c). In Montbéliarde and Normande, pedigree-based kinship coefficients seemed to plateau during both PTS and GS, although we observed a drop in the average pedigree-based kinship in Montbéliarde in 2008 (see Fig. 2c). The mean value of pedigree-based kinship coefficients was significantly lower for Montbéliarde born between 2012 and 2015 than for Montbéliarde bulls born between 2005 and 2010 (*p*-value < 1e−10), as well as for Normande bulls (*p*-value < 1e−10) (see Fig. 2c). By contrast, this value was significantly higher for Holstein bulls born between 2012 and 2015 than for those born between 2005 and 2010 (*p*-value < 1e−10) (see Fig. 2c).

#### Measures of genetic diversity based on runs of homozygosity (ROH)

The rate of ROH-based inbreeding increased significantly in Holstein under GS (between 2012 and 2015) compared to under PTS (between 2005 and 2010), i.e. from 0.080% per year to 0.55% per year for Holstein (from 0.39% to 1.36% per generation) with a relative change of 5.93 (see Table 2). This was not the case for Montbéliarde and Normande, for which inbreeding rates of 0.17 and 0.12% per year were found between 2005 and 2010, respectively (0.96 and 0.71% per generation) (see Table 2). In 2005, the overall ROH-based inbreeding level in Montbéliarde was significantly lower than that in Normande and Holstein (*p*-values = 0.0031 and 2.1e−08, respectively) (see Fig. 2d). However, in 2015, the level of ROH-based inbreeding was significantly higher in Holstein than in Montbéliarde and Normande (*p*-values < 1e−10, in both cases) (see Fig. 2d). The slopes of ROH-based inbreeding coefficients during GS are equivalent to increases of ~ 0.8, 1.4 and 5.4% per year of the mean ROH-based inbreeding coefficients in 2012 for Montbéliarde, Normande and Holstein, respectively.

Mean ROH length increased at a significantly higher rate in Holstein under GS (between 2012 and 2015) than under PTS (between 2005 and 2010), with a relative change of 7.77 (see Table 2). This was not the case in Montbéliarde and in Normande, for which the mean ROH length plateaued under GS (see Fig. 2e). From 2005 to 2015, the overall mean ROH length for Montbéliarde was significantly shorter than that for Normande, which in turn was significantly shorter than that for Holstein (*p*-values < 1e−10 in all cases, with average mean ROH lengths of 1.68, 1.71 and 1.80 Mb, respectively) (see Fig. 2c). The slopes of mean ROH lengths during GS showed increases of ~ 0.005, 0.07 and 0.7% per year of the mean ROH lengths in 2012 for Montbéliarde, Normande and Holstein, respectively.

#### Correlations between and variability of pedigree- and ROH-based inbreeding coefficients

Pearson’s product-moment correlation between pedigree- and ROH-based inbreeding coefficients were equal to 59% (95% confidence interval: [55%, 62%]), 50% [45%, 55%] and 59% [57%, 61%] for Montbéliarde, Normande and Holstein, respectively.

Coefficients of variation of pedigree-based inbreeding were equal to 0.33, 0.25 and 0.31 for Montbéliarde, Normande and Holstein, respectively. Coefficients of variation of ROH-based inbreeding were equal to 0.22, 0.18 and 0.21 for Montbéliarde, Normande and Holstein, respectively.

### Demographic parameters

#### Generation intervals

**Fig. 4:**
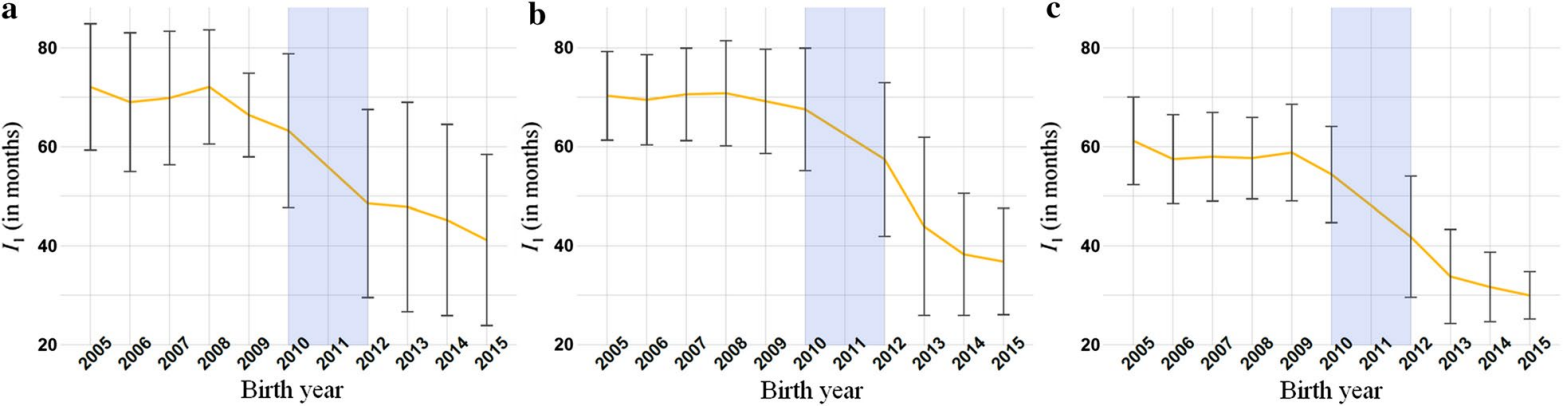
Parental generation intervals *I*_*1*_ (in months). *I*_*1*_ were defined as the difference between the birthdate of an individual and its parents’ (in months). The yellow line represents the mean for each birth year and the error bars the standard deviation. Generation intervals *I*_1_ were computed for progeny-tested bulls born between 2005 and 2010 and for marketed bulls born between 2012 and 2015, for Montbéliarde (**a**), Normande (**b**) and Holstein (**c**)

The mean difference $I_{1}$ between the birthdate of an individual and its parents reached a plateau at ~ 70 months in Montbéliarde and in Normande, and 59 months in Holstein between 2005 and 2009. $I_{1}$ was reduced by a factor 1.7 in Montbéliarde, by 1.9 in Normande and by a factor 2 in Holstein between animals born between 2005 and 2009 (under PTS) and animals born in 2015 (under GS). To date, this interval has not reached a new plateau for the three breeds (see Fig. 4).

#### Number and use of bulls

**Fig. 5:**
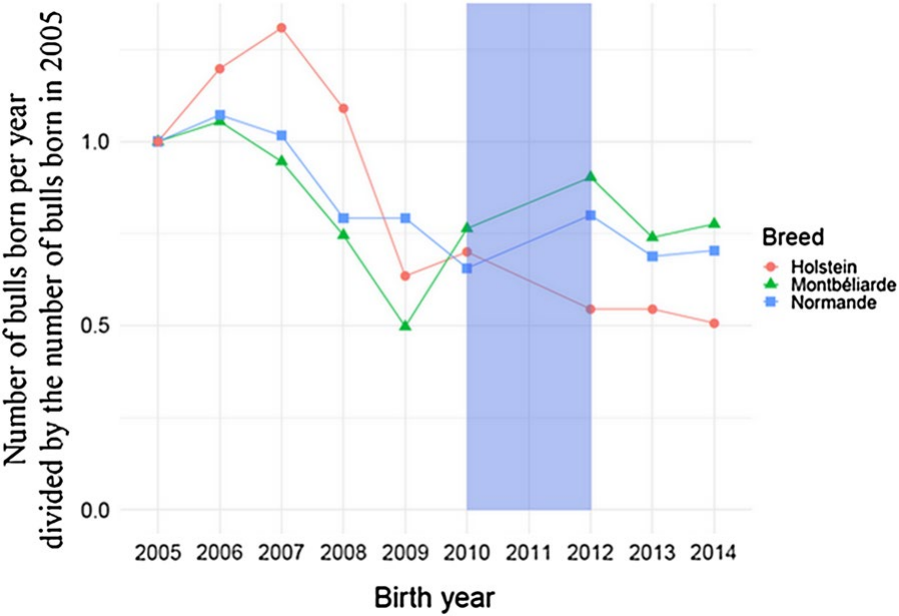
Proportion of bulls born each year in comparison with 2005. Proportions were computed for progeny-tested bulls born between 2005 and 2010 and for marketed bulls born between 2012 and 2015, for Montbéliarde (green), Normande (blue) and Holstein (red)

A histogram of the proportion of the total number of offspring per bull for each breed and selection type is available in Additional file 6: Figure S8 and Additional file 7: Table S6. The ratio between number of marketed bulls born in 2014 and number of progeny-tested bulls born in 2005 was equal to 77.6% (128 compared to 165) in Montbéliarde, 70.4% (88 compared to 125) in Normande, and 50.7% (294 compared to 580) in Holstein (see Fig. 5).

**Fig. 6:**
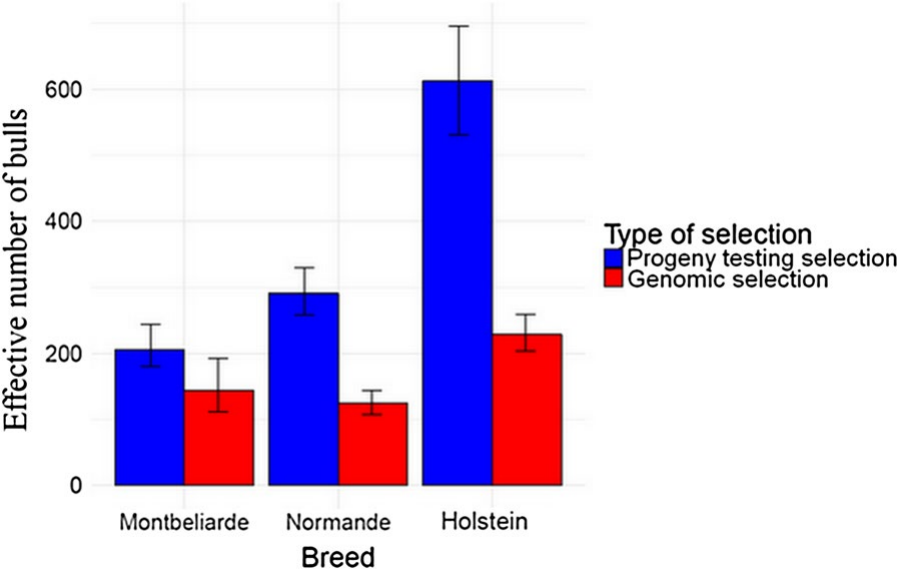
Effective number of bulls of three French dairy cattle breeds. Effective number of bulls were computed for progeny-tested bulls born between 2005 and 2010 for progeny testing selection (blue) and for marketed bulls born between 2012 and 2015 for genomic selection (red) for Montbéliarde, Normande and Holstein. Black bars represent 95% confidence intervals, generated by random resampling with replacement of the datasets (bootstrap with 1000 iterations)

**Fig. 7:**
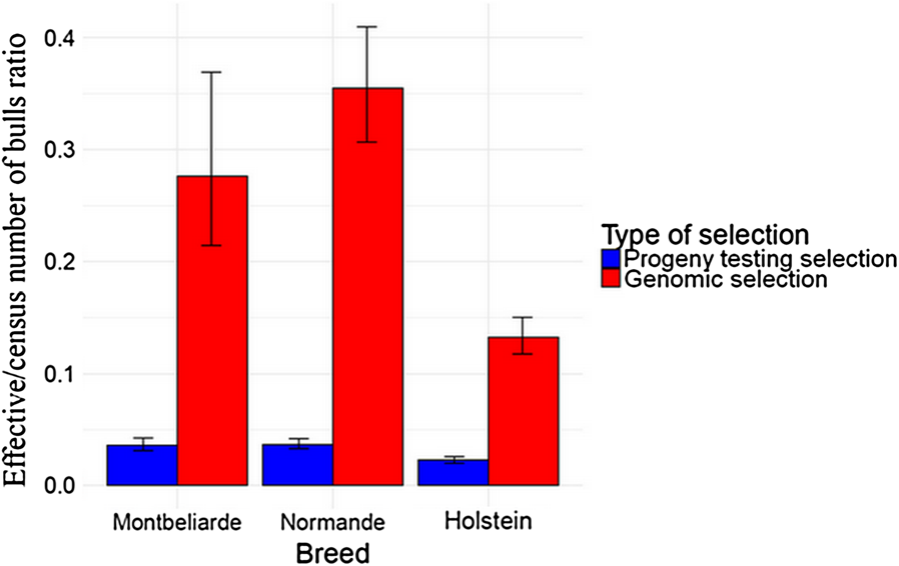
Effective/census number of bulls ratios of three French dairy cattle breeds. Effective number of bulls/Number of bulls ratios ${{Ne_{\text{breed, selection}} } \mathord{\left/ {\vphantom {{Ne_{\text{breed, selection}} } {n_{\text{breed, selection}} }}} \right. \kern-0pt} {n_{\text{breed, selection}} }}$ were computed for progeny-tested bulls for individuals born between 2005 and 2010 for progeny testing selection (blue) and for marketed bulls born between 2012 and 2015 for genomic selection (red) for Montbéliarde, Normande and Holstein. Black bars represent 95% confidence intervals, generated by random resampling with replacement of the datasets (bootstrap with 1000 iterations)

The effective number of bulls used was significantly larger between 2005 and 2010 (under PTS) than between 2012 and 2014 (under GS) in Normande and Holstein, but not in Montbéliarde (see Fig. 6). The ratio of the effective and the actual number of bulls ratio under PTS, $\frac{{Ne_{\text{breed, PTS}} }}{{n_{\text{breed, PTS}} }}$, was significantly lower than the same ratio under GS, $\frac{{Ne_{\text{breed, GS}} }}{{n_{\text{breed, GS}} }}$, in Montbéliarde, Normande and Holstein, with $\frac{{Ne_{\text{breed, GS}} }}{{n_{\text{breed, GS}} }}$ of 0.009, 0.021 and 0.003%, respectively (see Fig. 7).

## Discussion

In dairy cattle breeds, GS was expected to increase the annual genetic gain by up to 70 to 108% depending on the scenarios used for the breeding schemes (with different proportions of young genotypes bulls and proven bulls used) [1, 2, 44] but only a few studies have examined its impact on genetic diversity. These studies were preliminary and mainly predictive since they were conducted before GS had really started. Genomic evaluations were implemented in 2009 in French dairy cattle breeds [4] and they have drastically changed dairy cattle breeding schemes for the three major French dairy cattle breeds. Thus, after a decade of GS, we can evaluate the impact of new breeding schemes on both the genetic gain and diversity in these three breeds in France. The Holstein, Montbéliarde and Normande breeds in France vary in population size with 628,000, 319,000, 2,402,000 cows, respectively, as of January 2018 [45]. These breeds differ also in terms of breeding program: the Holstein breed is widespread and genetic material is exchanged globally (*i.e.* it is an international breed), whereas Montbéliarde and Normande are French typical breeds managed at the country level (*i.e.* national breeds). Thus, the results drawn from our study can compare these two situations. To conduct this study, we focused on bulls of these three breeds born between 2005 and 2010 for PTS, and bulls born between 2012 and 2015 for GS.

An increase in annual genetic gain with GS has been observed in most dairy cattle populations. For instance, in the US Holstein cattle breed, the annual genetic gain for milk, fat and protein yield in cows born before or after 2010 has increased by 71, 111 and 81%, respectively, between 2008 and 2014 [46, 47]. This can be explained by a drastic decrease in the generation interval by ~ 50% on average for Holstein bulls [10, 46]. This effective decrease in generation interval is consistent with previous predictions [9] and is mainly due to the withdrawal of progeny testing prior to marketing of bulls.

We obtained similar results for Montbéliarde, Normande and Holstein bulls. Their mean annual genetic gains (ΔG) increased by 50, 71 and 33%, respectively, after implementation of GS. At the same time, the bulls’ parental generation intervals were reduced by a factor 1.7, 1.9 and 2 for the Montbéliarde, Normande and Holstein breeds, respectively, suggesting that, similar to the US Holstein situation, most of the extra genetic gain is due to reduced generation intervals. However, there are differences between breeds in terms of increase in annual genetic gain and decrease in generation interval, with the Holstein breed having less increase in gain relative to the reduction in generation interval. This may be due to different formulas used for total merit indices and different breeding goals.

Given this global increase in genetic gain for all the breeds studied here along with the decrease in generation interval, one might be concerned about the trend in genetic diversity. Indeed, on the one hand, an accelerated decrease in genetic diversity could be expected due to the decrease in generation interval. On the other hand, genomic evaluations can lead to a more diverse offer in terms of candidates for selection, and thus a moderation of inbreeding rate over time [5]. To study the possible change of inbreeding rate, we first looked at various estimates of inbreeding and diversity to recognize whether and why they differed. ROH-based inbreeding estimates aligned well with pedigree-based inbreeding estimates when averaged per breed. However, pedigree-based inbreeding estimates had a higher coefficient of variation between bulls within a breed (0.33, 0.25 and 0.31 for Montbéliarde, Normande and Holstein, respectively) than ROH-based inbreeding estimates (0.22, 0.18 and 0.21 for Montbéliarde, Normande and Holstein, respectively), for the three breeds. Pedigree depths were substantial and similar in the three breeds and there was limited variation among individual bulls; the mean number of generations traced for Montbéliarde, Normande and Holstein bulls was 15.4 ± 1.7, 15.8 ± 1.6 and 15.4 ± 1.9, respectively. Therefore, pedigree completeness was not a major cause of the higher coefficient of variation of pedigree-based inbreeding estimates in comparison with ROH-based inbreeding estimates. Pedigree-based estimations of individual inbreeding coefficients are based on expected IBD probabilities and because of Mendelian sampling these values might differ from ROH-based estimates, which reflects more the realized or effective inbreeding [48]. The correlations between pedigree- and ROH-based inbreeding estimates ranged from 50 to 59%, depending on the breed. These correlations were in the range of those previously reported for Jersey and Danish red cattle (52 and 54%, respectively) [49] but lower than those reported for Holstein (around 80%) [49] and simulated cattle (around 70%) [11]. Errors and depth of pedigree are very plausible reasons for these differences between reported and presented correlations. Moreover, such differences in these correlations are expected because pedigree-based inbreeding corresponds to the expected value of inbreeding while ROH-based inbreeding corresponds to its realized value. In groups of highly selected bulls, linkage disequilibrium and homozygosity can be strong, which leads to large discrepancies between expected (based on pedigree) and observed (based on ROH) inbreeding and subsequently lower correlations.

In all three breeds, the ratio between the effective and actual number of bulls (Ne/N) increased after implementation of GS. This reflects changes in breeding schemes in all three breeds, which should allow for a decrease in inbreeding rate and more generally for a slowdown in the annual loss of genetic diversity. However, we distinguished two contrasting types of behavior of inbreeding based on ROH. First, for the two national breeds, Montbéliarde and Normande, we did not detect any significant change in the annual inbreeding rate (ΔF). Second, for the Holstein international breed there was a significant increase in ΔF, which has increased from 0.080% to 0.55% per year based on ROH, and from 0.088% to 0.49% per year based on pedigree. This result does not align with the fact that all three breeds experienced a significant increase in annual genetic gain, with the lowest increase observed in Holstein while the inbreeding rate in Holstein was highest. This result suggests that changes in breeding schemes reflected by an increase of Ne/N of bulls in Holstein did not allow for a slowdown in the annual loss of genetic diversity.

For Holstein, ΔF per generation also increased from 0.39% to 1.36% based on ROH, and from around 0.46% to 1.39% based on pedigree. Thus, the decrease in generation interval did not explain all the increase in ΔF per year for Holstein, since ΔF per generation increased too. The observed inbreeding rate in Holstein is higher than the 1% per generation acceptable rate of inbreeding according to FAO guidelines [50]. Since the accumulation of inbreeding through ROH is not linear, and the observed level and rate of inbreeding could be underestimated when based on ROH, the inbreeding problem is likely even more severe than suggested by these figures.

We used a linear regression to estimate the rate of ROH-based inbreeding because it gave better results than a logarithmic regression in terms of residuals of the model (see Additional file 4: Figures S1–S7). When estimated from pedigree data, the slope of inbreeding increased significantly for both Normande and Holstein bulls, reaching 0.19% and 0.49% per year, respectively. The inbreeding rate reached 0.70% and 1.39% per generation, respectively, so the decrease in generation interval did not explain all the increase in inbreeding rate per year. Thus, the Normande breed remained under the recommended FAO threshold whereas Holstein exceeded this when estimating inbreeding on pedigree data as well as on ROH data. Why there are such differences between these two breeds should be further investigated, and in particular, the differences in breeding schemes, such as the use of foreign bulls or the setting-up of quotas for the number of inseminations allowed per bull.

From 2005 to 2015, the overall mean ROH length for Montbéliarde (1.68 Mb) was significantly shorter than that for Normande (1.71 Mb), which in turn was significantly shorter than that for Holstein (1.80 Mb). The length of ROH depends on two main criteria, for given detection parameters and genotyping density: (i) the effective population size and the level of linkage disequilibrium [51]; and (ii) the number of generations that separates an individual from the ancestor from which the ROH originated, with longer ROH reflecting more recent inbreeding [28, 52]. We investigate these two possibilities to explain the differences in mean ROH lengths between breeds.

Estimated effective population sizes (Ne) of French dairy cattle breeds were equal to 79 for Montbéliarde, 87 for Normande and 96 for Holstein in 2017 [53]. Based on these results, effective/census population sizes ratios (Ne/N) of 0.009, 0.021 and 0.003% for these breeds, respectively, were calculated [53]. The effective number of bulls born under GS for the period between 2012 and 2015 was estimated to be 144 for Montbéliarde, 124 for Normande and 229 for Holstein, and effective/census number of bull ratios were 28, 35 and 13%, respectively. In both cases, the ratios between effective and census effective population sizes were lowest for Holstein and higher for Normande and Montbéliarde. We can hypothesize that these differences are probably linked to the small numbers of bulls that have strong contributions and the low ratios between effective and census number of bulls, particularly in Holstein [54]. Moreover, whereas the distribution of the number of offspring per bull became less skewed for the three breeds under GS than under PTS, this change seemed to be less pronounced in Holstein (see Additional file 6: Figure S8, Additional file 7: Table S6). This was consistent with a relatively low ratio between effective and census number of bulls in Holstein under GS in comparison with Montbéliarde and Normande, and with the hypothesis of a remaining effect of a few bulls with strong contributions. There can be two effects explaining the greater observed ROH length for Holstein. The first is the small effective population size relative to census population size resulting in a highly structured genome in this breed with long haploblocks [55] where recombination is unlikely to have occurred leading to long ROH. The second is recent inbreeding, likely to have occurred because of the massive use of few elite bulls. This was suggested by kinship estimated from pedigree being higher for the period between 2012 and 2015 than for the period between 2005 and 2010 for Holstein and not for national breeds, which can be explained by the intensive use of a few individuals as grandfathers of bulls, mostly coming from the USA [56]. We chose not to compute kinship from GRM because of their high sensitivity to the estimation of allelic frequencies (data not shown) [21]. The hypothesis that a few individuals were used as grandfathers of bulls in Holstein was consistent with the increase by a factor 8.77 (with a relative change of 7.77) of the annual rate of mean ROH length in Holstein breed after the beginning of GS, whereas it remained unchanged in the Montbéliarde and Normande breeds. This increase might be counterintuitive. Indeed, GS may lead to relatively shorter ROH than PTS [10]. Therefore, the increase in mean ROH length in Holstein might be underestimated and was corroborated by the increase in the number of long ROH per individual in Holstein with the beginning of GS, whereas it was not the case in Montbéliarde and Normande (see Additional file 8: Table S7, Additional file 9: Figures S9–S11]). Therefore, the increase in the number of long ROH per individual in Holstein might be responsible for the increased mean ROH length within the breed. Shorter ROH tend to result from older inbreeding (older common ancestors) whereas longer ROH tend to result from more recent inbreeding (more recent common ancestors) [29, 31, 32]. Thus, the increase in the mean ROH length and in the number of long ROH in Holstein suggests an increase in the number of ROH segments arising from recent inbreeding. This introduction of recent inbreeding was confirmed by the observed increase in pedigree-based inbreeding calculated from the last five generations that started when GS was implemented, with an inbreeding rate ranging from − 0.083% per year for PTS to 0.27% per year for GS (− 0.40% and 0.78% per generation, respectively). In conclusion, in Holstein, this increase in the mean ROH length might reflect the substantial introduction of recent inbreeding, but also a reduction in the number of selected bulls.

## Conclusions

After the introduction of genomic selection, the annual genetic gain increased based on estimated breeding values for bulls of three large French dairy cattle breeds: Montbéliarde, Normande and Holstein. At the same time, the inbreeding rate per year and per generation increased in Holstein bulls, an international breed, but not in Montbéliarde and Normande bulls, two national breeds. Small effective population sizes resulted in highly structured genomes with long haploblocks, particularly in Holstein, as a consequence of the low effective/census number of bulls ratios. A joint increase in genetic gain and conservation of genetic diversity is possible by (i) applying optimal contribution selection; (ii) carrying out on-farm mating plans; and (iii) ensuring that these tools are flexible enough to be used effectively in the field. Our study highlights the fact that changes in breeding schemes can impact genetic diversity, and thus, it is important to continue monitoring genetic diversity and to study the future impact of different breeding schemes strategies on genetic diversity. The recent implementation of genomic evaluations in small regional breeds, for which effective population size can be limited, should be studied carefully in order to ensure sustainability of breeding schemes in the future, in terms of both genetic gain and diversity. The spread of new technologies in breeding schemes, such as female reproductive technologies and embryo transfer that might increase global selection intensity through the dams of bulls, represents a risk in terms of genetic diversity that should be anticipated in the light of our conclusions.

## Supplementary information

**Additional file 1: Table S1.** Description of the three studied dairy cattle breeds: Montbéliarde, Normande and Holstein.

**Additional file 2: Table S2.** Length of the autosomal genome in three French dairy cattle breeds. Length of autosomal genome on which ROH can be detected corresponds to the length of the autosomal genome covered by SNPs after withholding gaps longer than 150 kb between two SNPs. The proportion of autosomal genome on which ROH can be detected is the length of autosomal genome on which ROH can be detected divided by the total length of the autosomal genome (from *Bos taurus* assembly 3.1.1).

**Additional file 3: Table S3.** Parental generation intervals. Mean parental generation intervals for bulls born between 2005 and for bulls born between 2010 and 2015 in months and in years, for Montbéliarde, Normande and Holstein.

**Additional file 4: Figure S1.** Square root of standardized residuals for the linear regression of pedigree-based inbreeding depending on birth year (Y5) and selection type (Gen) for Montbéliarde, Normande and Holstein (from left to right). **Figure S2.** Square root of standardized residuals for the linear regression of pedigree-based inbreeding for the last five generations depending on birth year (Y5) and selection type (Gen) for Montbéliarde, Normande and Holstein (from left to right). **Figure S3.** Square root of standardized residuals for the linear regression of pedigree-based kinship depending on birth year (Y5) and selection type (Gen) for Montbéliarde, Normande and Holstein (from left to right). **Figure S4.** Square root of standardized residuals for the linear regression of *F*_ROH_, inbreeding based on ROH depending on birth year (Y5) and selection type (Gen) for Montbéliarde, Normande and Holstein (from left to right). **Figure S5.** Square root of standardized residuals for the logarithmic regression of *F*_ROH_, inbreeding based on ROH depending on birth year (Y5) and selection type (Gen) for Montbéliarde, Normande and Holstein (from left to right) (*F*_ROH, transformed_ = log10(1−*F*_ROH_)). **Figure S6.** Square root of standardized residuals for the linear regression of the mean length of ROH depending on birth year (Y5) and selection type (Gen) for Montbéliarde, Normande and Holstein (from left to right). **Figure S7.** Square root of standardized residuals for the linear regression of total merit index (ISU) depending on birth year (Y5) and selection type (Gen) for Montbéliarde, Normande and Holstein (from left to right).

**Additional file 5: Table S4.** Pedigree-based inbreeding calculated from the last five generations in percent per year. **Table S5.** Estimation of the slopes of pedigree-based inbreeding calculated from the last five generations in percent per generation.

**Additional file 6: Figure S8.** Proportion of the total number of offspring per bull for each breed and selection type. Progeny testing selection corresponds to bulls born between 2005 and 2010 and genomic selection to bulls born between 2012 and 2014.

**Additional file 7: Table S6.** Number of bulls and offspring for each birth period in the dataset.

**Additional file 8: Table S7.** ROH categories lengths thresholds.

**Additional file 9: Figure S9.** Confidence intervals of the number of ROH per ROH length category per selection type for Montbéliarde bulls. **Figure S10.** Confidence intervals of the number of ROH per ROH length category per selection type for Normande bulls. **Figure S11.** Confidence intervals of the number of ROH per ROH length category per selection type for Holstein bulls.
