# Supplementary material for: The impact of genomic selection on genetic diversity and genetic gain in three French dairy cattle breeds
Source: Genet Sel Evol. 2019 Sep 23;51:52. doi: 10.1186/s12711-019-0495-1 (PMC6757367; doi:10.1186/s12711-019-0495-1)
Supplement: Supplementary file 4 — Additional file 4: Figure S1. Square root of standardized residuals for the linear regression of pedigree-based inbreeding depending on birth year (Y5) and selection type (Gen) for Montbéliarde, Normande and Holstein (from left to right). Figure S2. Square root of standardized residuals for the linear regression of pedigree-based inbreeding for the last five generations depending on birth year (Y5) and selection type (Gen) for Montbéliarde, Normande and Holstein (from left to right). Figure S3. Square root of standardized residuals for the linear regression of pedigree-based kinship depending on birth year (Y5) and selection type (Gen) for Montbéliarde, Normande and Holstein (from left to right). Figure S4. Square root of standardized residuals for the linear regression of FROH, inbreeding based on ROH depending on birth year (Y5) and selection type (Gen) for Montbéliarde, Normande and Holstein (from left to right). Figure S5. Square root of standardized residuals for the logarithmic regression of FROH, inbreeding based on ROH depending on birth year (Y5) and selection type (Gen) for Montbéliarde, Normande and Holstein (from left to right) (FROH, transformed = log10(1−FROH)). Figure S6. Square root of standardized residuals for the linear regression of the mean length of ROH depending on birth year (Y5) and selection type (Gen) for Montbéliarde, Normande and Holstein (from left to right). Figure S7. Square root of standardized residuals for the linear regression of total merit index (ISU) depending on birth year (Y5) and selection type (Gen) for Montbéliarde, Normande and Holstein (from left to right). [file 12711_2019_495_MOESM4_ESM.docx]

**Additional file 4: Residuals of regressions of genetic diversity and genetic gain parameters depending on birth year and selection type.**

**Figure S1: Square root of standardized residuals for the linear regression of pedigree-based inbreeding depending on birth year (Y5) and selection type (Gen) for Montbéliarde, Normande and Holstein (from left to right)**

**Figure S2: Square root of standardized residuals for the linear regression of pedigree-based inbreeding for the last five generations depending on birth year (Y5) and selection type (Gen) for Montbéliarde, Normande and Holstein (from left to right)**


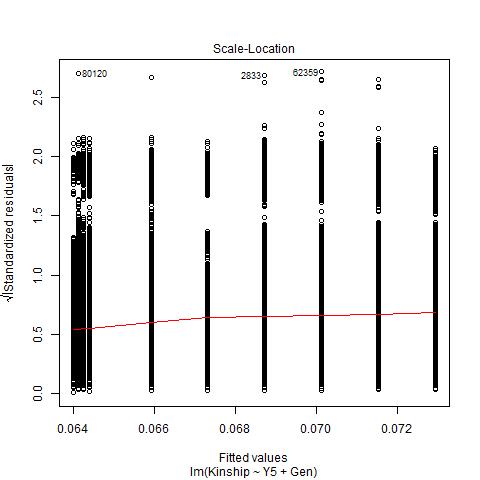

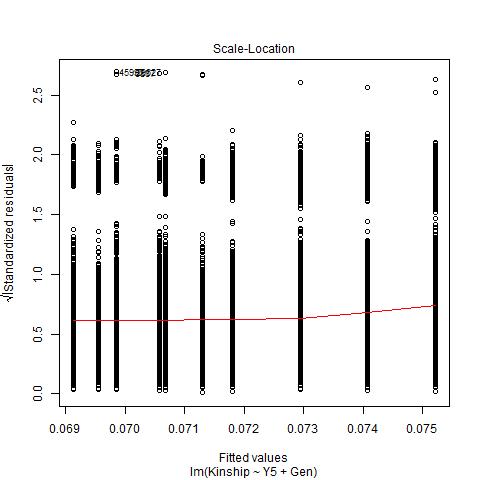

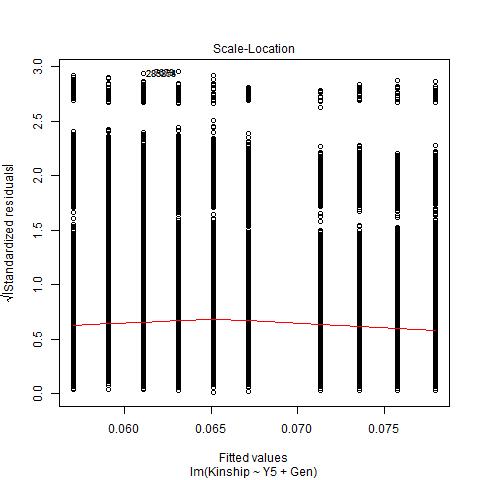


**Figure S3: Square root of standardized residuals for the linear regression of pedigree-based kinship depending on birth year (Y5) and selection type (Gen) for Montbéliarde, Normande and Holstein (from left to right)**

**Figure S4: Square root of standardized residuals for the linear regression of *F*_ROH_, inbreeding based on Runs of Homozygosity (ROH) depending on birth year (Y5) and selection type (Gen) for Montbéliarde, Normande and Holstein (from left to right)**

**Figure S5: Square root of standardized residuals for the logarithmic regression of *F*_ROH_, inbreeding based on Runs of Homozygosity (ROH) depending on birth year (Y5) and selection type (Gen) for Montbéliarde, Normande and Holstein (from left to right)**

*F*_ROH, transformed_ = log10(1-*F*_ROH_)

**Figure S6: Square root of standardized residuals for the linear regression of the mean length of Runs of Homozygosity (ROH) depending on birth year (Y5) and selection type (Gen) for Montbéliarde, Normande and Holstein (from left to right)**

**Figure S7: Square root of standardized residuals for the linear regression of Total Merit Index (ISU) depending on birth year (Y5) and selection type (Gen) for Montbéliarde, Normande and Holstein (from left to right)**
