# Supplementary material for: The impact of genomic selection on genetic diversity and genetic gain in three French dairy cattle breeds
Source: Genet Sel Evol. 2019 Sep 23;51:52. doi: 10.1186/s12711-019-0495-1 (PMC6757367; doi:10.1186/s12711-019-0495-1)
Supplement: Supplementary file 5 — Additional file 5: Table S4. Pedigree-based inbreeding calculated from the last five generations in percent per year. Table S5. Estimation of the slopes of pedigree-based inbreeding calculated from the last five generations in percent per generation. [file 12711_2019_495_MOESM5_ESM.docx]

**Additional file 5: Pedigree-based inbreeding calculated from the last five generations per year and per generation.**

**Table S4: Pedigree-based inbreeding calculated from the last five generations in percent per year.**

| Breed | *b*_1_ (± standard error) | *p*-value of *b_1_* | $\delta$ (± standard error) | *p*-value of $\delta$ | *b_2_* | Relative change |
| --- | --- | --- | --- | --- | --- | --- |
| Montbéliarde | -0.16 (± 0.030) | < 2e-16 | -0.16 (± 0.077) | 0.039 | -0.32 | -1.00 |
| Normande | -0.030 (± 0.024) | < 2e-16 | -0.20 (± 0.059) | 7.4e-04 | -0.23 | -6.65 |
| Holstein | -0.083 (± 0.013) | 9.6e-11 | 0.36 (± 0.032) | < 2.2e-16 | 0.27 | 4.30 |

b_1_ is the slope of regression of pedigree-based inbreeding calculated from the last five generations depending on birth year for progeny tested bulls born between 2005 and 2010 (progeny testing selection), and δ the difference between the slopes of regression of pedigree-based inbreeding calculated from the last five generations depending on birth year for progeny testing selection and for marketed bulls born between 2012 and 2015 (genomic selection), for Montbéliarde, Normande and Holstein. The relative change is equal to$\frac{\delta}{\left| b_{1} \right|}$. b_2_ is the slope of the pedigree-based inbreeding for the last five generations for marketed bulls born between 2012 and 2015 (genomic selection), equal to b_1_ +$\delta$. The p-value of $\delta$ corresponds to the significance of the non-nullity of δ.

**Table S5: Estimation of the slopes of pedigree-based inbreeding calculated from the last five generations in percent per generation.**

| Breed | *b*_1_ per generation | $\delta$ per generation | *b_2_* per generation | Relative change per generation |
| --- | --- | --- | --- | --- |
| Montbéliarde | -0.91% | -3.1e-03 | **-1.22%** | -0.34 |
| Normande | -0.18% | -6.9e-03 | **-0.86%** | -3.94 |
| Holstein | -0.40% | 1.2e-02 | **0.78%** | 2.95 |
