# Supplementary material for: The impact of genomic selection on genetic diversity and genetic gain in three French dairy cattle breeds
Source: Genet Sel Evol. 2019 Sep 23;51:52. doi: 10.1186/s12711-019-0495-1 (PMC6757367; doi:10.1186/s12711-019-0495-1)
Supplement: Supplementary file 6 — Additional file 6: Figure S8. Proportion of the total number of offspring per bull for each breed and selection type. Progeny testing selection corresponds to bulls born between 2005 and 2010 and genomic selection to bulls born between 2012 and 2014. [file 12711_2019_495_MOESM6_ESM.docx]

**Additional file 6: Proportion of the total number of offspring per bull for each breed and selection type.**

**Figure S8: Proportion of the total number of offspring per bull for each breed and selection type**

Progeny testing selection corresponds to bulls born between 2005 and 2010 and genomic selection to bulls born between 2012 and 2014.

For example, for Montbéliarde, around 50% of the bulls born under genomic selection (y-axis) are the fathers of less than 0.003 = 0.3% of all calves (x-axis) and around 30% of the bulls are the fathers of 0.3% to 0.6% of all calves.
