# Supplementary material for: The impact of genomic selection on genetic diversity and genetic gain in three French dairy cattle breeds
Source: Genet Sel Evol. 2019 Sep 23;51:52. doi: 10.1186/s12711-019-0495-1 (PMC6757367; doi:10.1186/s12711-019-0495-1)
Supplement: Supplementary file 7 — Additional file 7: Table S6. Number of bulls and offspring for each birth period in the dataset. [file 12711_2019_495_MOESM7_ESM.docx]

**Additional file 7: Number of bulls and offspring for each birth period in the dataset.**

**Table S6: Number of bulls and offspring for each birth period in the dataset.**

| Breed | Birth period | Total number of individuals | Total number of offspring |
| --- | --- | --- | --- |
| Montbéliarde | 2005 – 2010  Progeny-tested bulls | 826 | 1,102,266 |
|  | 2012 – 2014  Marketed bulls | 399 | 562,178 |
| Normande | 2005 – 2010  Progeny-tested bulls | 666 | 542,939 |
|  | 2012 – 2014  Marketed bulls | 347 | 437,558 |
| Holstein | 2005 – 2010  Progeny-tested bulls | 3440 | 4,573,893 |
|  | 2012 – 2014  Marketed bulls | 1248 | 2,390,778 |
