# Supplementary material for: The impact of genomic selection on genetic diversity and genetic gain in three French dairy cattle breeds
Source: Genet Sel Evol. 2019 Sep 23;51:52. doi: 10.1186/s12711-019-0495-1 (PMC6757367; doi:10.1186/s12711-019-0495-1)
Supplement: Supplementary file 8 — Additional file 8: Table S7. ROH categories lengths thresholds. [file 12711_2019_495_MOESM8_ESM.docx]

**Additional file 8: ROH categories lengths thresholds**

For each breed, we selected bulls born in 2005, obtaining three cohorts: Montbéliarde bulls born in 2005, Normande bulls born in 2005 and Holstein bulls born in 2005. For each of these three cohorts, five ROH length categories were defined with R function kmeans (R Core Team, 2018) by applying it to the list of all ROH for all individuals of each cohort, for each breed separately. We obtained length thresholds for each category.

**Table S7: ROH categories lengths thresholds**

| Category | Montbéliarde | | Normande | | Holstein | |
| --- | --- | --- | --- | --- | --- | --- |
|  | Minimum ROH length (kb) | Maximum ROH length (kb) | Minimum ROH length (kb) | Maximum ROH length (kb) | Minimum ROH length (kb) | Maximum ROH length (kb) |
| 1 | 1000.079 | 1426.286 | 1000.051 | 1458.437 | 1000.079 | 1465.802 |
| 2 | 1426.639 | 2080.933 | 1459.128 | 2121.894 | 1466.58 | 2163.376 |
| 3 | 2084.642 | 3046.285 | 2127.988 | 3087.975 | 2164.072 | 3247.136 |
| 4 | 3055.704 | 4723.705 | 3091.578 | 4730.121 | 3249.867 | 5031.288 |
| 5 | 4749.088 | 8718.715 | 4761.585 | 8217.436 | 5040.034 | 9126.138 |

**REFERENCES**

R Core Team (2018). R: A language and environment for statistical computing. R Foundation for Statistical Computing, Vienna, Austria. URL https://www.R-project.org/.
