# Supplementary material for: The impact of genomic selection on genetic diversity and genetic gain in three French dairy cattle breeds
Source: Genet Sel Evol. 2019 Sep 23;51:52. doi: 10.1186/s12711-019-0495-1 (PMC6757367; doi:10.1186/s12711-019-0495-1)
Supplement: Supplementary file 9 — Additional file 9: Figure S9. Confidence intervals of the number of ROH per ROH length category per selection type for Montbéliarde bulls. Figure S10. Confidence intervals of the number of ROH per ROH length category per selection type for Normande bulls. Figure S11. Confidence intervals of the number of ROH per ROH length category per selection type for Holstein bulls. [file 12711_2019_495_MOESM9_ESM.docx]

**Additional file 9: Number of ROH in different length categories**

For each breed, we selected bulls born in 2005, obtaining three cohorts: Montbéliarde bulls born in 2005, Normande bulls born in 2005 and Holstein bulls born in 2005. For each of these three cohorts, five ROH length categories were defined with R function kmeans (R Core Team, 2018) by applying it to the list of all ROH for all individuals of each cohort, for each breed separately. We obtained length thresholds for each category [see Additional file 8 Table S7]. Then, we counted how many ROH were in each category for each individual. This allowed computing the mean number of ROH per individual per category for all individuals born during progeny testing selection (between 2005 and 2010) or during genomic selection (between 2012 and 2015) for each breed separately. We then performed an anova to compare the number of ROH in each category between progeny testing and genomic selection and estimated confidence intervals of these numbers with R function emmeans (Russell, 2018).

*

*

*

**Figure S9: Confidence intervals of the number of ROH per ROH length category per selection type for Montbéliarde bulls.** *: mean ROH numbers were significantly different between progeny testing and genomic selection for this category.


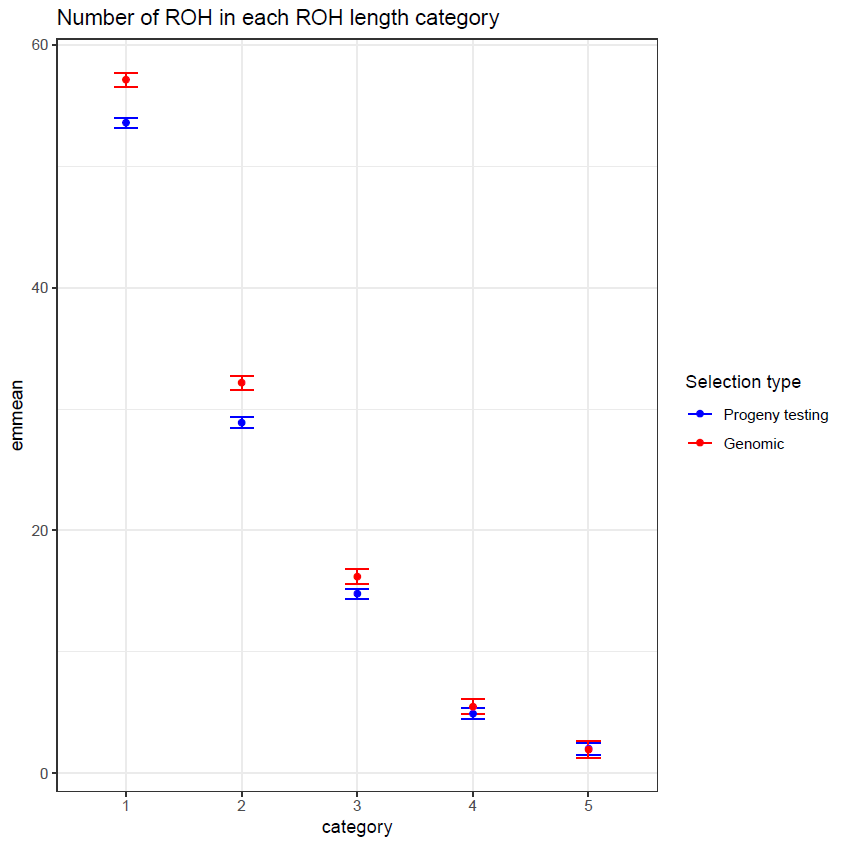


*

*

*

**Figure S10: Confidence intervals of the number of ROH per ROH length category per selection type for Normande bulls.** *: mean ROH numbers were significantly different between progeny testing and genomic selection for this category.

*

*

*

*

**Figure S11: Confidence intervals of the number of ROH per ROH length category per selection type for Holstein bulls.** *: mean ROH numbers were significantly different between progeny testing and genomic selection for this category.

In conclusion, small (categories 1 and 2) and medium length (category 3) ROH were significantly more numerous during genomic selection than during progeny testing selection for all three breeds. However, long ROH (category 4, between 3.25 and 5.03 Mb) were more significantly numerous during genomic selection than during progeny testing selection only for Holstein.

**REFERENCES**

R Core Team (2018). R: A language and environment for statistical computing. R Foundation for Statistical Computing, Vienna, Austria. URL https://www.R-project.org/.

Russell Lenth (2018). emmeans: Estimated Marginal Means, aka Least-Squares Means. R package version 1.3.0. https://CRAN.R-project.org/package=emmeans
